# Oral arsenic plus imatinib versus imatinib solely for newly diagnosed chronic myeloid leukemia: a randomized phase 3 trial with 5-year outcomes

**DOI:** 10.1007/s00432-024-05700-x

**Published:** 2024-04-11

**Authors:** Jie Tian, Yong-Ping Song, Gao-Chong Zhang, Shu-Fang Wang, Xiao-Xiang Chu, Ye Chai, Chun-Ling Wang, Ai-Li He, Feng Zhang, Xu-Liang Shen, Wei-Hua Zhang, Lin-Hua Yang, Da-Nian Nie, Dong-Mei Wang, Huan-Ling Zhu, Da Gao, Shi-Feng Lou, Ze-Ping Zhou, Guo-Hong Su, Yan Li, Jin-Ying Lin, Qing-Zhi Shi, Gui-Fang Ouyang, Hong-Mei Jing, Sai-Juan Chen, Jian Li, Jian-Qing Mi

**Affiliations:** 1 State Key Laboratory of Medical Genomics, Shanghai Institute of Hematology, National Research Center for Translational Medicine at Shanghai, Ruijin Hospital, Shanghai Jiao Tong University School of Medicine, Shanghai, China; 2 The Affiliated Cancer Hospital of Zhengzhou University, Zhengzhou, Henan China; 3 Yifan Research & Development, Hefei, Anhui China; 4 Lanzhou University Second Hospital, Lanzhou, Gansu China; 5 The Affiliated Huaian No 1 People’s Hospital of Nanjing Medical University, Huaian, Jiangsu China; 6 The Second Affiliated Hospital of Xi’an Jiaotong University, Xi’an, Shaanxi China; 7 The First Affiliated Hospital of Bengbu Medical College, Bengbu, Anhui China; 8 Heping Hospital Affiliated to Changzhi Medical College, Changzhi, Shanxi China; 9 The First Hospital of Shanxi Medical University, Taiyuan, Shanxi China; 10 The Second Hospital of Shanxi Medical University, Taiyuan, Shanxi China; 11 The Second Affiliated Hospital of Sun Yat-Sen University, Guangzhou, Guangdong China; 12 Hengshui People’s Hospital, Hengshui, Hebei China; 13 West China Hospital of Sichuan University, Chengdu, Sichuan China; 14 The Affiliated Hospital of Inner Mongolia Medical University, Hohhot, Inner Mongolia China; 15 The Second Affiliated Hospital of Chongqing Medical University, Chongqing, China; 16 The Second Affiliated Hospital of Kunming Medical University, Kunming, Yunnan China; 17 Cangzhou Central Hospital, Cangzhou, Hebei China; 18 The First Affiliated Hospital of China Medical University, Shenyang, Liaoning, China; 19 The People’s Hospital of Guangxi Zhuang Autonomous Region, Nanning, Guangxi China; 20 The Second Affiliated Hospital of Nanchang University, Nanchang, Jiangxi China; 21 Ningbo First Hospital, Ningbo, Zhejiang China; 22 Peking University Third Hospital, Beijing, China

**Keywords:** Chronic myeloid leukemia, Arsenic, Imatinib, Efficacy and safety

## Abstract

**Purpose:**

The synergistic effects of combining arsenic compounds with imatinib against chronic myeloid leukemia (CML) have been established using in vitro data. We conducted a clinical trial to compare the efficacy of the arsenic realgar–indigo naturalis formula (RIF) plus imatinib with that of imatinib monotherapy in patients with newly diagnosed chronic phase CML (CP-CML).

**Methods:**

In this multicenter, randomized, double-blind, phase 3 trial, 191 outpatients with newly diagnosed CP-CML were randomly assigned to receive oral RIF plus imatinib (n = 96) or placebo plus imatinib (n = 95). The primary end point was the major molecular response (MMR) at 6 months. Secondary end points include molecular response 4 (MR^4^), molecular response 4.5 (MR^4.5^), progression-free survival (PFS), overall survival (OS), and adverse events.

**Results:**

The median follow-up duration was 51 months. Due to the COVID-19 pandemic, the recruitment to this study had to be terminated early, on May 28, 2020. The rates of MMR had no significant statistical difference between combination and imatinib arms at 6 months and any other time during the trial. MR^4^ rates were similar in both arms. However, the 12-month cumulative rates of MR^4.5^ in the combination and imatinib arms were 20.8% and 10.5%, respectively (*p* = 0.043). In core treatment since the 2-year analysis, the frequency of MR^4.5^ was 55.6% in the combination arm and 38.6% in the imatinib arm (*p* = 0.063). PFS and OS were similar at five years. The safety profiles were similar and serious adverse events were uncommon in both groups.

**Conclusion:**

The results of imatinib plus RIF as a first-line treatment of CP-CML compared with imatinib might be more effective for achieving a deeper molecular response (Chinadrugtrials number, CTR20170221).

**Supplementary Information:**

The online version contains supplementary material available at 10.1007/s00432-024-05700-x.

## Introduction

*Chronic myeloid leukemia (CML)* is characterized by the ***BCR::ABL1*** oncoprotein, which produces a constitutively active tyrosine kinase that leads to pathogenesis (Heisterkamp et al. 1983). Tyrosine kinase inhibitors (TKIs) can inhibit the activity of the ***BCR::ABL1*** fusion protein to trigger cell apoptosis. They have shown high rates of molecular responses and improved prognoses in patients with chronic phase (CP) of chronic myeloid leukemia (CML) (Hochhaus et al. 2020). The major molecular response (MMR) is recognized as the main optimal milestone of CML treatment (Deininger et al. 2020; Hochhaus et al. 2020). Nevertheless, a deep molecular response (DMR, molecular response 4 or better), *particularly molecular response 4.5 (MR*^*4.5*^*) or more, has been recently regarded as the gateway to treatment discontinuation and treatment-free remission (TFR) for patients with CP-CML, resulting in a low risk of progression* (Deininger et al. 2020; Cortes et al. 2021). *However, the rate of MR*^*4.5*^* is only 1–11% with TKIs in 1 year and 8–25% in 2 years* (Hehlmann et al. 2014; Cortes et al. 2016, 2018; Hochhaus et al. 2016a, b; Hochhaus et al. 2016a, b). Therefore, it is crucial to identify other methods to increase the proportion of deeper molecular responses.

Intravenous arsenic trioxide and oral tetra-arsenic tetrasulfide (As_4_S_4_) have proven to be highly effective and safe and are used as a standard treatment for acute promyelocytic leukemia (APL) (Lu et al. 2002; Zhu et al. 2018). Based on in vitro data in the K562 cell line and CML primary cells from patients, arsenic can directly induce cell apoptosis and degrade ***BCR::ABL1*** rather than inhibiting ***BCR::ABL1*** activity as would a TKI (Li et al. 2002; Yin et al. 2004; Mao et al. 2010). Subsequently, additional results were observed in a mouse model of ***BCR::ABL***-positive CML, where *arsenic acted on ****BCR::ABL**** by directly binding the RING finger domain of c-CBL to inhibit its self-ubiquitination/degradation, while imatinib inhibits the PI3K/AKT/mTOR pathway* (Zhang et al. 2009; Mao et al. 2010; Liu et al. 2014). The synergistic effect of the two drugs at the molecular level might be a promising approach to improving response rates in CML patients.

In addition, the realgar–indigo naturalis formula (RIF), an oral arsenic agent (As_4_S_4_)-containing formula, is convenient for managing medication and not inferior to intravenous arsenic trioxide (Zhu et al. 2018).

Based on these findings, this study aimed to determine if RIF plus imatinib led to a higher and deeper confirmed molecular response compared to imatinib monotherapy. *This randomized controlled trial of arsenic combined with imatinib in CML treatment is the first of its kind and could potentially contribute to achieving higher TFR in CP*.

## Methods

### Patient recruitment

Eligible patients for the trial were aged between 18 and 75 years and diagnosed with CP Ph-positive CML within 12 months prior to study entry. CP-CML was defined as the presence of less than 15% blasts, less than 20% basophils, and less than 30% blasts plus promyelocytes in peripheral blood and bone marrow, as well as a platelet count of less than 100 × 10^9^ cells/L unrelated to therapy (Cortes et al. 2021). The participants did not harbor any extramedullary disease except for hepatosplenomegaly. Previous treatment for CML was excluded, with the exception of hydroxyurea and anagrelide. Further details on the inclusion and exclusion criteria are available in Online Resource 1. All participants provided written informed consent with documents approved by the institutional review board of each participating center, and the study was conducted following the Declaration of Helsinki.

### Trial design and randomization

*This was a randomized, double-blind, placebo-controlled, multicenter, phase 3 trial, conducted in an outpatient setting.* Participants who met the eligibility criteria were randomly assigned in a 1:1 ratio, using an interactive voice response system, to receive either RIF (provided by Yifan Pharmaceutical Co., Ltd.) in the combination with imatinib (Hansoh Pharma Co., Ltd.) or placebo (Yifan Pharmaceutical Co., Ltd.) plus imatinib. Stratified block randomization was applied according to the Sokal risk score (Sokal et al. 1984) at the time of diagnosis. The participants and study staff were blinded to the treatments in this double-blind trial.

### Procedures

Following randomization, all participants received imatinib daily. Participants assigned to the combination group received RIF, and those in the imatinib group received a placebo (RIF simulant) for 14 consecutive days every month. The total treatment duration was 12 months (a month defined as 28 days). After 12 months, all the participants received imatinib daily. The RIF (270 mg per tablet) contained realgar (As_4_S_4_, 30 mg per tablet), indigo naturalis (125 mg per tablet), Radix salviae miltiorrhizae (50 mg per tablet), Radix pseudostellariae (45 mg per tablet), and a collagen capsule (20 mg per tablet). Details of the treatment procedures, dose modifications, and laboratory assessments are provided in Online Resource 1.

### Evaluation of efficacy

The primary endpoint was to compare the significant molecular response (MMR) (Hochhaus et al. 2020) rate at 6 months. MMR was defined as ***BCR::ABL1***^*IS*^ transcript level of 0.1% or lower.

Secondary endpoints included MMR rates at 3, 9, and 12 months; molecular response 4 (MR^4^) and MR^4.5^ rates at 3, 6, 9, and 12 months; times to MMR, MR^4^, and MR^4.5^; progression-free survival (PFS); and overall survival (OS). MR^4^ and MR^4.5^ were defined as ***BCR::ABL1***^*IS*^ transcript levels ≤ 0.01% or ≤ 0.0032%, respectively (Cortes et al. 2021). Treatment failure was defined as no complete hematologic response and/or Ph^+^ > 95% at 3 months, ***BCR::ABL1*** > 10% and/or Ph^+^ > 35% at 6 months, ***BCR::ABL1*** > 1% and/or Ph^+^ > 0 at 12 months, and loss of complete hematologic remission, loss of complete cytogenetic response, loss of MMR, or clonal chromosomal abnormalities in Ph^+^ cells at any time (Baccarani et al. 2013). PFS was defined as progression to the accelerated phase (AP), blastic phase (BP), or CML-related death at any time since randomization. OS was defined as the time from randomization to death caused by any cause at any time, including during follow-up and after discontinuation of the study treatment. Trial visits were conducted at screening, baseline, and 3, 6, 9, 12, 18, 24, 48, and 60 months.

### Safety analysis

The safety analyses included all patients who received at least one dose of any study medication. The participants were analyzed according to the dose of any study drug administered at the start of treatment. Adverse events (AEs) were graded according to the Common Terminology Criteria for AEs (version 4.03) of the National Cancer Institute (https://evs.nci.nih.gov/ftp1/CTCAE/CTCAE_4.03/CTCAE_4.03_2010-06-14_QuickReference_8.5×11.pdf).

### Statistical analysis

*This study was designed to test the primary hypothesis that RIF plus imatinib for newly diagnosed CP-CML was superior to imatinib alone, with an estimation of increment by 12% of absolute improvement in MMR at 6 months, i.e., an estimated increase from 18% (imatinib) to 30% (combination treatment). The estimate of 18% of patients achieving an MMR at 6 months with imatinib was based on a previously reported, randomized, first-line study (*Cortes et al. 2018*). The original sample size had been computed with a type 1 error rate of 0.05 and 80% power. We calculated that after adjusting for 20% dropout, the total planned sample size was 496 participants. However, only 191 participants were finally enrolled due to the COVID-19 pandemic in 2020.*

All the efficacy analyses were performed based on the intention-to-treat (ITT) principle. In addition, a per-protocol (PP) analysis was conducted. Response rates were tested at a significance level of 0.05 using the chi-square test or Fisher’s exact test. Cumulative response rates, PFS, and OS were estimated using the Kaplan–Meier method, and groups were compared using the log-rank test. All reported* P* values were two-sided. Nominal two-sided *P* values were provided for descriptive purposes only, without multiplicity adjustment.

Safety was analyzed for all participants who received at least one dose of the assigned treatment. Data were analyzed using the SPSS software (version 25). The study was registered at www.chinadrugtrials.org.cn (number, CTR20170221).

## Results

### Participants

**Fig. 1:**
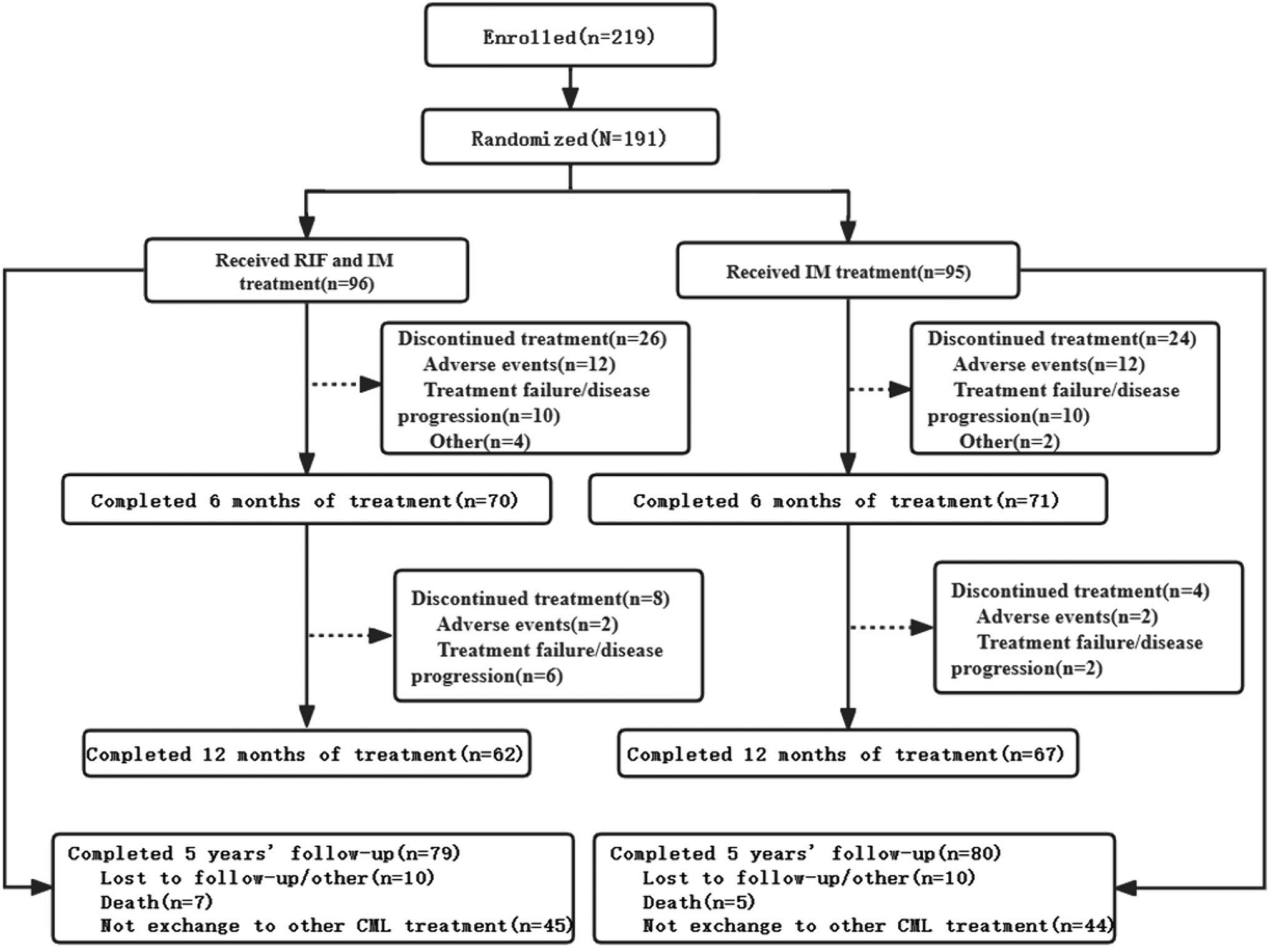
CONSORT diagram for this trial after a minimum follow-up of 5 years (data cutoff 11 November 2022). During the 12-month randomized, double-blind, placebo-controlled trial, all the patients remained unaware of their trial-group assignments. An analysis of ***BCR::ABL1*** transcript level was performed every 3 months. Of the 219 patients with chronic myeloid leukemia who underwent screening, 191 were enrolled, 129 completed the trial and 159 completed a 5-year follow-up

From May 2017 to May 2020, 219 newly diagnosed CP-CML cases were assessed for eligibility at 21 study centers in China (Fig. 1). Of these, 191 underwent randomization and were included in the ITT population. *The combination group (RIF plus imatinib) consisted of 96 patients who were assigned to this treatment arm and 95 were assigned to the imatinib group (placebo plus imatinib)*. The two groups were well balanced in terms of sex, age, and Sokal score. The key baseline and clinical characteristics are summarized in Table 1. In May 2020, because of the pandemic, the sponsor initiated the closure of the trial recruitment. This recommendation was approved by an independent data monitoring committee. Therefore, the current analysis is based on data collected up to November 11, 2022.

**Table 1:** Demographic and baseline characteristics, modified intent-to-treat population

	RIF/IM (*n* = 96)	Placebo/IM (*n* = 95)	Total (*n* = 191)
Age			
Median (range), years	46 (18–74)	50 (23–71)	48 (18–74)
Median time from diagnosis to random assignment, days (range)*	9 (0–104)	8 (0–375)	8 (0–375)
Sex (*n*, %)			
Male	56 (58.3)	52 (54.7)	108 (56.5)
Sokal risk group (*n*, %)			
Low	45 (46.9)	44 (46.3)	89 (46.6)
Intermediate	38 (39.6)	38 (40.0)	76 (39.8)
High	13 (13.5)	13 (13.7)	26 (13.6)
ECOG performance status (*n*, %)			
0	81 (84.4)	80 (84.2)	161 (84.3)
1 ~ 2	15 (15.6)	15 (15.8)	30 (15.7)
Prior CML therapy (*n*, %)^†^			
Yes	85 (88.5)	76 (80.0)	161 (84.3)
Hydroxyurea	83 (86.5)	75 (78.9)	158 (82.7)
rINFα	1 (1.0)	0	1 (0.5)
Leukapheresis	4 (4.2)	1 (1.1)	5 (2.6)
History of disease (*n*, %)^‡^			
Yes	70 (72.9)	67 (70.5)	137 (71.7)
Cardiovascular disease (*n*, %)	25 (26.0)	20 (21.1)	45 (23.6)
Hypertension	19 (19.8)	14 (14.7)	33 (17.3)
Coronary disease	0	1 (1.1)	1 (0.5)
Hyperlipidemia	3 (3.1)	6 (6.3)	9 (4.7)
Diabetes	6 (6.3)	4 (4.2)	10 (5.2)
Other heart disease	1 (1.0)	3 (3.2)	4 (2.1)

Data are no. (%) unless noted otherwise. The modified intent-to-treat population includes Philadelphia chromosome-positive patients with typical ***BCR::ABL1*** transcript types

*RIF* realgar–indigo naturalis formula, *IM* imatinib, *ECOG* Eastern Cooperative Oncology Group, *CML* chronic myeloid leukemia

*Defined as time from primary diagnosis to random assignment

^†^Prior therapy for CML included hydroxyurea and/or rINFα treatment only as permitted by eligibility criteria; no prior treatment with a tyrosine kinase inhibitor was allowed

^‡^Per case report form collected at screening

A total of 157 participants (82.2%) completed the 12-month double-blind intervention (Table 2). The median dose was 1620 mg per day (range, 810–1890 mg, including 90–210 mg realgar) for RIF in the combination arm and 400 mg/day (range, 300–400 mg/day) for imatinib in both treatment arms. The median duration of treatment was 12.0 months (range, 1.1–12.0 months) for combination therapy and 12.0 months (range, 1.5–12.0 months) for imatinib. After 5 years, as of the data cutoff, 83.3% of combination-treated patients and 83.2% of imatinib-treated participants were still on follow-up (Table 2 and Supplementary Table S2). The median follow-up times were 51 months (range, 0–65 months) and 50 months (range, 0–65 months) for the RIF arm and 51 months (range, 2–65 months) for the imatinib arm at the time of analysis. The percentage of participants who continued imatinib treatment after the study was similar between the RIF group (46.9%, *n* = 45) and the imatinib group (46.3%, *n* = 44). Thirty-five participants in both study arms received other anti-CML drugs (Supplementary Table S2).

**Table 2:** Treatment status of study patients, safety population

Treatment status	RIF/IM (n = 96)	Placebo/IM (n = 95)
Remaining on study*	78 (81.3)	79 (83.2)
Completed 12 months of treatment	62 (64.6)	67 (70.5)
Discontinued treatment within 12 months	34 (35.4)	28 (29.5)
Discontinued treatment within 6 months	26 (27.1)	24 (25.3)
Related to study treatment	16 (16.7)	12 (12.6)
Not related to study treatment	18 (18.8)	16 (16.8)
Disease progression to AP/BP*	1 (1.0)	1 (1.1)
Adverse event	14 (14.6)	14 (14.7)
Adverse event within 6 months	12 (12.5)	12 (12.6)
Serious adverse event	3 (3.1)	2 (2.1)
Suboptimal response/treatment failure	16 (16.7)	12 (12.6)
Investigator request	2 (2.1)	0
Patient request	1 (1.0)	1 (1.1)
Death	0	0
Lost to follow-up	1 (1.0)	1 (1.1)
Other	1 (1.0)	0

Data are no. (%). The safety population includes all patients who received one or more doses of study treatment

*RIF* realgar–indigo naturalis formula, *IM* imatinib, *AP* accelerated phase, *BP* blast phase

*Patients on treatment or in follow-up (safety or survival) from randomization to 12 months

### Efficacy

#### Primary endpoint

*The rates of an MMR at 6 months in the ITT population were 11.5% in the combination arm and 12.6% in the imatinib arm (****p*** = *0.828).* In the PP population, the rate was 14.3% in the combination arm and 17.3% in the imatinib arm (***p*** = 0.768). No statistically significant differences existed between the groups.

#### Secondary endpoints

**Fig. 2:**
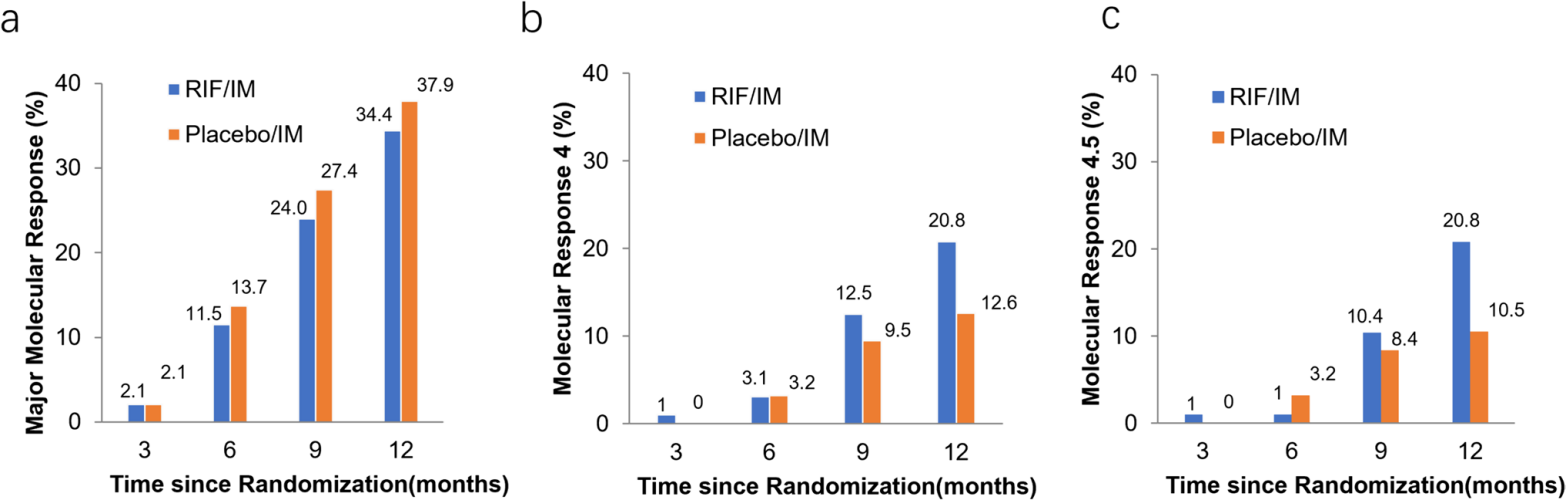
Cumulative molecular response rates. The results in the intention-to-treat population were calculated by means of the Cochran–Mantel–Haenszel test, stratified according to the Sokal risk group, after the last patient had completed 12 cycles of therapy (with a 28-day duration for each cycle). Cumulative proportion of patients with **a** major molecular response (MMR; ***BCR::ABL***^*IS*^ ≤ 0.1%), **b** molecular response 4 (MR^4^; ***BCR::ABL***^*IS*^ ≤ 0.01%), and **c** molecular response 4.5 (MR^4.5^; ***BCR::ABL***^*IS*^ ≤ 0.0032%) by 3, 6, 9, and 12 months is shown. *IS* international scale

**Fig. 3:**
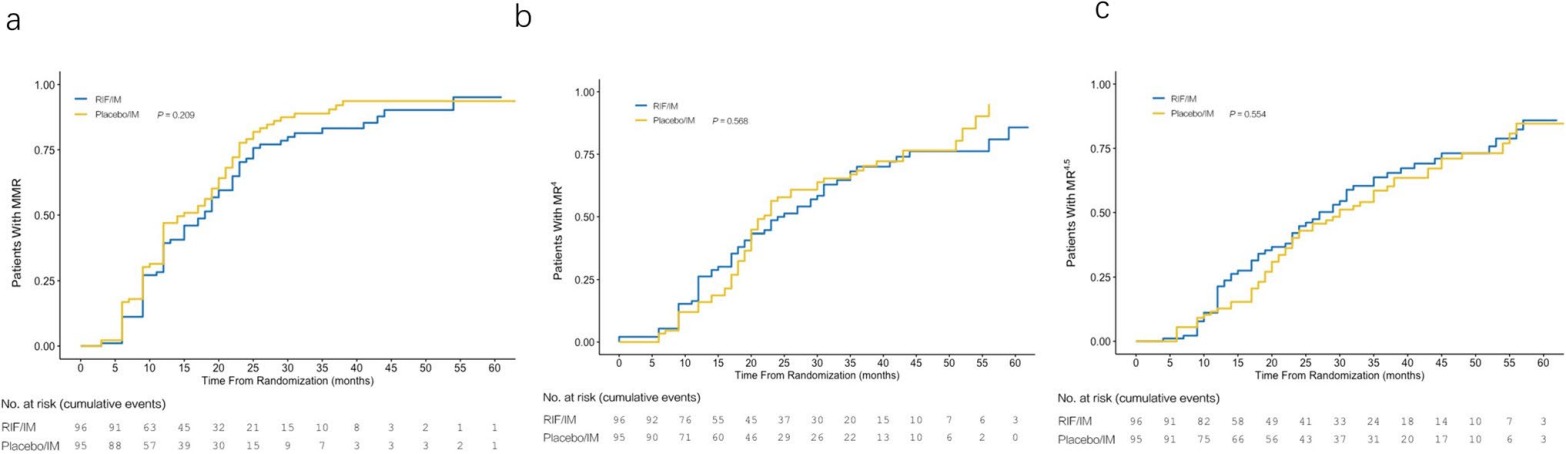
Cumulative response rates over time. The percentages of patients with **a** major molecular response (MMR; ***BCR::ABL***^*IS*^ ≤ 0.1%), **b** molecular response 4 (MR^4^; ***BCR::ABL***^*IS*^ ≤ 0.01%), and **c** molecular response 4.5 (MR^4.5^; ***BCR::ABL***^*IS*^ ≤ 0.0032%) by 1, 2, 3, 4, and 5 years are shown. *IS* international scale

In the ITT analysis, the rates of MMR at 3, 6, 9, and 12 months were similar between the two groups (Fig. S1a), as well as the rates of MR^4^ and MR^4.5^ at 3, 6, 9, and 12 months (Fig. S1b and c). *No significant difference between MR*^*4*^* or MR*^*4.5*^* at 3, 6, or 9 months among patients of trial groups was observed. However, from the 9th month onward, there was increasingly improved efficacy with combination treatment compared with imatinib treatment* (Fig. S1 and Fig. 2). Moreover, by 12 months, MR^4^ was achieved/maintained in 20.8% of participants receiving combination treatment and in 12.6% of participants receiving imatinib (***p*** = 0.129). *The cumulative rate of MR*^*4.5*^* by 12 months was 10.3% higher with combination treatment than imatinib monotherapy (20.8% vs. 10.5%, respectively, ****p*** = *0.043) in ITT populations (*Fig. 3*).*

In the PP analysis, the data of MMR and MR^4^ showed no significant differences between the two groups (Fig. S2). However, the cumulative rate of MR^4.5^ at 12 months in the combination treatment group was higher than in imatinib monotherapy one (32.3% vs. 14.9%, ***p*** = 0.020) (Fig. S3).

At the data cutoff after 5 years, 45 (46.9%) and 44 (46.3%) participants in the combination and imatinib arms, respectively, remained on core treatment (without change to other anti-CML drugs); 79 (82.3%) and 80 (84.2%) randomized participants remained in the study (either on treatment or follow-up after discontinuation of study treatment; Supplementary Table S2). The cumulative rates of participants (ITT population) with responses in the final analysis (5 years follow-up) in the combination and imatinib arms were 72.9% and 76.8% for MMR (***p*** = 0.209), 61.5% and 61.1% for MR^4^ (*p* = 0.568), and 61.5% and 58.9% for MR^4.5^ (*p* = 0.554), respectively (Fig. 3). *In the 2-year analysis* of participants *on core treatment, the frequency of MR*^*4.5*^* in the combination arm tended to be higher compared with the imatinib arm (55.6% vs. 38.6%, p* = *0.063)*.

**Fig. 4:**
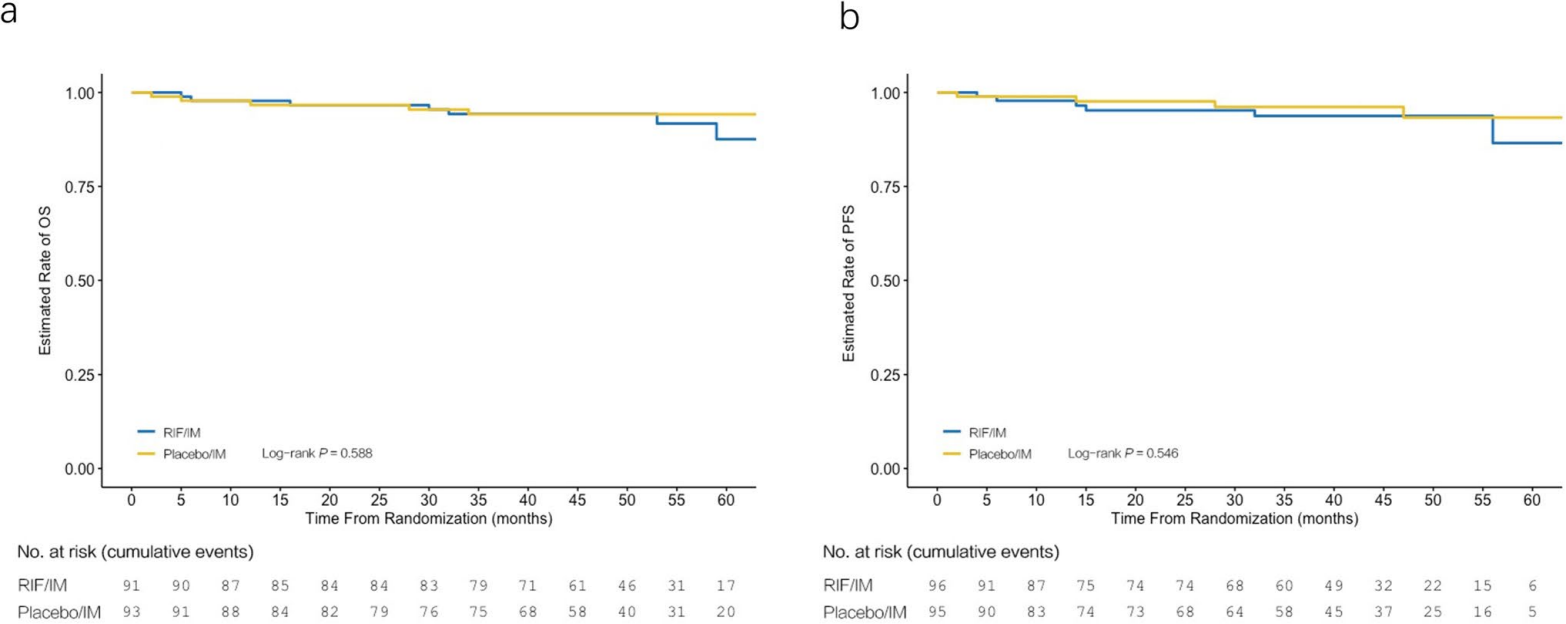
Kaplan–Meier curves for **a** overall survival (OS) and **b** progression-free survival (PFS) in both treatment arms. OS and PFS were calculated using patients on study treatment and in follow-up after discontinuation of randomly assigned treatment

*By 5 years, PFS was 93.7% in the combination arm and 95.8% in the imatinib arm* (*p* = 0.546, Fig. 4). Progression of CML to AP/BP was similar at 12 months, occurring in one participant in each group. One in the combination arm occurred at five months after randomization, and the other in the imatinib arm occurred at two months. Based on the data cutoff, the number of patients with AP/BP was six (6.3%) in the combination arm and four (4.2%) in the imatinib arm.

*By 5 years, the OS rate was not statistically significantly different between study groups (92.3% for combination therapy and 94.6% for imatinib, p* = *0.588*, Fig. 4). Based on the data cutoff, the number of deaths was seven in the combination arm and five in the imatinib arm, and the number of CML-related deaths was four and two, respectively (Supplementary Table S2).

The achievement of MR^4.5^ by 12 months was also investigated using the Sokal score (Supplementary Fig. S4). A significant difference between combination- and imatinib-treated participants with confirmed molecular assessment was observed for MR^4.5^ in the intermediate-risk group (*p* = 0.035, Supplementary Fig. S4). Five-year outcomes according to Sokal risk scores were also similar (Supplementary Table S3).

### Safety

The safety population comprised 191 participants who received at least one dose of the study drug. Both trial treatments were safe and consistent with the known safety AE profiles. *The overall rates of drug-related AEs associated with both treatments were similar* (combination therapy, 96.9%; imatinib, 91.6%), and grade 3 or grade 4 events were observed (combination therapy, 35.4%; imatinib, 40.0%). Grade 3 or grade 4 non-hematological AEs were uncommon in all patients (6.3% in each group; Table 3). *Of the nonhematologic adverse drug reactions occurring in the treated participants, the combination therapy, when compared with imatinib, was associated with higher incidences of diarrhea (27.1% vs. 14.7%, p* = *0.036), vomiting (26.0% vs. 17.9%, p* = *0.004), and nausea (34.4% vs. 17.9%, p* = *0.006) with significant difference between trial groups. These were primarily grade 1 or grade 2 events.*

**Table 3:** Safety findings

Event	RIF/IM (*n* = 96)		Placebo/IM (*n* = 95)	
	Any grade (*n*, %)	Grade 3/grade 4 (*n*, %)	Any grade (*n*, %)	Grade 3/grade 4 (*n*, %)
Nonhematologic adverse event	88 (91.7)	6 (6.3)	83 (87.4)	6 (6.3)
Rash	6 (6.3)	0 (0.0)	13 (13.7)	2 (2.1)
Pruritus	9 (9.4)	0 (0.0)	12 (12.6)	0 (0.0)
Diarrhea	26 (27.1)	0 (0.0)	14 (14.7)	0 (0.0)
Vomiting	25 (26.0)	1 (1.0)	17 (17.9)	0 (0.0)
Nausea	33 (34.4)	1 (1.0)	17 (17.9)	1 (1.1)
Muscle ache	25 (26.0)	0 (0.0)	32 (33.7)	0 (0.0)
Muscle cramps	2 (2.1)	0 (0.0)	12 (12.6)	0 (0.0)
Abdominal pain	19 (19.8)	0 (0.0)	12 (12.6)	0 (0.0)
Abdominal distension	14 (14.6)	0 (0.0)	9 (9.5)	0 (0.0)
Fatigue	14 (14.6)	0 (0.0)	10 (10.5)	0 (0.0)
Edema*	65 (67.7)	2 (2.1)	57 (60.0)	0 (0.0)
Facial edema	58 (60.4)	2 (2.1)	50 (52.6)	0 (0.0)
Pyrexia	12 (12.5)	0 (0.0)	13 (13.7)	1 (1.1)
Upper respiratory infection	38 (39.6)	1 (1.0)	28 (29.5)	0 (0.0)
Bleeding	5 (5.2)	0 (0.0)	11 (11.6)	1 (1.1)
Cytopenia	71 (74.0)	30 (31.3)	62 (65.3)	26 (27.4)
Anemia	43 (44.8)	5 (5.2)	36 (37.9)	7 (7.4)
Thrombocytopenia	34 (35.4)	15 (15.6)	38 (40.0)	13 (13.7)
Neutropenia	64 (66.7)	23 (24.0)	52 (54.7)	17 (17.9)
Laboratory abnormality	59 (61.5)	4 (4.2)	52 (54.7)	5 (5.3)
Elevated ALT	32 (33.3)	0 (0.0)	12 (12.6)	1 (1.1)
Elevated AST	30 (31.3)	0 (0.0)	10 (10.5)	1 (1.1)
Elevated ALP	15 (15.6)	0 (0.0)	16 (16.8)	0 (0.0)
Elevated GGT	8 (8.3)	0 (0.0)	10 (10.5)	1 (1.1)
Hyperglycemia	7 (7.3)	0 (0.0)	13 (13.7)	0(0.0)
Elevated LDH	7 (7.3)	0 (0.0)	11 (11.6)	0 (0.0)
Hypophosphatemia	25 (26.0)	3 (3.1)	19 (20.0)	2 (2.1)

Data are number of patients (%). Adverse events were graded according to the Common Toxicity Criteria of the National Cancer Institute, and their occurrence in at least 10% of either group is reported. No deaths (grade 5 events) were recorded that were thought to be related to treatment

*RIF* realgar–indigo naturalis formula, *IM* imatinib, *ALT* alanine aminotransferase, *AST* aspartate aminotransferase, *ALP* alkaline phosphatase, *GGT* γ-glutamyl transpeptidase

*Edema includes edema, eye edema, orbital and periorbital edema, face edema, localized edema, edema peripheral, allergic edema, weight increase, pharyngeal edema, and pulmonary edema

Diarrhea was frequently reported in the first month of treatment, and the median time to onset was 18 days (range: 1–294 days) in the combination arm compared with 35 days (range: 2–172 days) in the imatinib arm. Vomiting occurred frequently in the first 3 months after starting treatment in both groups, and the median time to onset was 7 days (range, 1–240 days) with the combination treatment and 18 days (range, 1–301 days) with imatinib. The median time to nausea onset was 6 days (range, 0–480 days) in the combination group and 2 days (range, 0–67 days) in the imatinib group.

*Conversely, rash occurred less frequently with combination treatment than with imatinib, as did pruritus, muscle ache, bleeding, and muscle cramps; only muscle cramps (p* = *0.003)* were significantly different between the trial groups.

Many participants in both arms experienced multiple cytopenia events (combination arm, 74.0%; imatinib arm, 65.3%). Grade 3 or grade 4 neutropenia occurred in 24.0% of the patients in the combination arm and 17.9% of the patients in the imatinib arm. However, the incidences of grade 3 or grade 4 anemia and thrombocytopenia were similar between the treatment arms (Table 3). The median time to onset of grade 3 or grade 4 hematologic AEs was 79 days (range, 35–104 days) for the combination treatment and 45 days (range, 29–70 days) for imatinib, with median durations of 28 and 21 days, respectively.

The most frequent biochemical abnormalities are listed in Table 3. Grade 3 or grade 4 biochemical laboratory abnormalities were observed in 4.2% of the participants receiving combination therapy and 5.3% of those receiving imatinib. *The grades at any level were similar in both groups*. In the combination arm, *the incidence of elevated alanine aminotransferase (ALT) and aspartate aminotransferase (AST) was more than two times than that in the imatinib arm (combination arm, 33.3%, and 31.3%; imatinib arm, 12.6%, and 10.5%, respectively, p* = *0.006 for ALT and p* < *0.001 for AST)*; however, with regard to grade 3 or grade 4 elevation of ALT and AST levels, no participant was found in the combination group, and only one case was observed in the imatinib group. Additional data are presented in Tables S3 and S4.

Drug-related cardiac AEs such as prolonged QT interval, sinus bradycardia, and auricular premature beat, most presenting as grade 1 or grade 2, were experienced by 12 participants receiving combination therapy and 10 participants receiving imatinib. QT prolongation occurred only in the imatinib group (n = 4, one participant with grade 3). Additional data are available in Tables S4 and S5 in the Supplementary Appendix.

The causes of treatment discontinuation were AE, disease progression to AP/BP, loss to follow-up, and others (Table 2), with AE being the most prevalent reason (46.7%, *n* = 28). Discontinuation of treatment caused by AEs was 12.5% in the combination arm and 12.6% in the imatinib arm and most frequently occurred within 6 months of treatment (Table 2). *Serious AEs were uncommon in both arms. Of those participants who experienced dose interruptions and/or reductions for gastrointestinal symptoms, the rate was 8.3% for combination treatment and 2.1% for imatinib monotherapy (p* = *0.053); for hepatic toxic effects, it was higher on treatment among participants treated with combination therapy versus imatinib monotherapy (10.4% vs. 3.2%, p* = *0.046)* (Table 3). Dose interruptions and/or reductions in hematological abnormalities were common in both groups (combination arm, 24.0%; imatinib arm, 22.1%). Most AEs were controllable, and most participants who experienced dose interruptions and/or reductions took the trial medication again. No new safety signals were observed in either group during the 5-year follow-up, and no participant died because of adverse events. *Overall, the combination therapy was well tolerated and side effects were controllable.*

## Discussion

Arsenic is a standard treatment agent for APL and has a long history of use in CML. It was the standard treatment for CML before the emergence of chemotherapy in the late nineteenth century (Forkner and Scott 1931, Zhu et al. 2018). Our study was based on several previous studies that had confirmed the synergistic effects of RIF and imatinib in CML cells and mice (Li et al. 2002; Yin et al. 2004; Zhang et al. 2009; Mao et al. 2010). *While As*_*4*_*S*_*4*_* triggers ****BCR::ABL1**** degradation, imatinib inhibits its tyrosine kinase activity.* The combination of these two agents was found to be able to lower protein and enzymatic activity levels of ***BCR::ABL*** (Yin et al. 2004). Arsenic considerably could upregulate c-CBL, which serves as an E3 ligase for several receptor/protein tyrosine kinases, including ***BCR::ABL***, and mediate the ubiquitination and degradation of ***BCR::ABL*** (Mao et al. 2010).

In this randomized phase 3 trial, we compared the arsenic-containing combination therapy, RIF plus imatinib, with the current standard first-line therapy, imatinib, as an initial trial for newly diagnosed CP-CML. The recruitment of this study was terminated earlier than the initial design because of the coronavirus COVID-19 pandemic. Of the planned enrollment of 488 participants, only 191 participants were recruited; 157 participants reached the 12-month time point for analysis of the molecular response, and 159 participants were still on follow-up at 5 years.

In this trial, the primary endpoint of MMR at six months of combination therapy was similar to imatinib monotherapy based on the ITT population. The MMR rate in the imatinib arm at 12 months was 35.8%, which is consistent with that in other randomized trials (22–36.9%) (Kantarjian et al. 2010; Saglio et al. 2010; Wang et al. 2015; Cortes et al. 2018). *No significant differences were observed in MMR and MR*^*4*^* rates between the two groups at any time within 12 months. However, there was a trend toward higher rates of achieving these endpoints with combination therapy compared to imatinib alone. A significant improvement in MR*^*4.5*^* with combination therapy versus imatinib was identified at 12 months (p* = *0.043), although the efficacy analyses were limited by early termination of recruitment.* Moreover, *at 2 years since randomization, 16.9% more participants who received combination therapy achieved MR*^*4.5*^* than those who received imatinib alone (55.6% vs. 38.6%).* In recent years, several studies have confirmed that the depth of a patient’s molecular response is positively associated with the probability of TFR success and is essential for determining whether TFR is achieved (Hochhaus et al. 2017b, Takahashi et al. 2018a, b; Takahashi et al. 2018a, b). However, the *5-year MMR, MR*^*4*^*, and MR*^*4.5*^* rates were similar in the treatment groups. This could be owing to the longer duration of the effect by imatinib required against CML-initiating cells or alternative treatment with the second-generation TKIs after discontinuing the study treatment*.

Current therapeutic aims are directed at achieving sufficient DMR to reduce the risks of blast crisis transformation and increase the rates of TFR, which have been extensively investigated and are now part of the management of CML patients (Deininger et al. 2020; Stuckey et al. 2020; Cortes et al. 2021; Krishnan et al. 2022). Etienne et al. reported that 233 patients treated with front-line imatinib who achieved MR^4.5^ had better event-free survival and failure-free survival than those with a complete cytogenetic response and MMR status (Etienne et al. 2014). More consensus has been reached that sustained MR^4.5^ is an ideal objectif and is associated with higher TFR rates than sustained MR^4^ (Etienne et al. 2014; Branford 2020; Deininger et al. 2020; Cortes et al. 2021). In those trials on the second-generation TKIs in patients with newly diagnosed CP-CML, the cumulative rates of MR^4.5^ by 12 months with dasatinib (DASISION trial), bosutinib (BFORE trial), nilotinib 300 mg twice daily, and nilotinib 400 mg twice daily (ENESTnd trial) were 5%, 6.4%, 7%, and 11%, respectively, and after 5 years were 33%, 46%, 52%, and 54%, respectively (Cortes et al. 2016, 2018; Hochhaus et al. 2016a, b; Brümmendorf et al. 2022), which were superior to that with imatinib. Although comparisons between trials should be considered with caution, the *MR*^*4.5*^* rate with RIF plus imatinib (20.8% by 12 months) may be potentially equal to or even higher than observed with the second-generation TKIs.* Several TKI discontinuation trials with limited long-term follow-ups have been reported. TFR rates in imatinib discontinuation studies for patients with a sustained MR^4.5^ or better were estimated as 47%–65% at 12 months and 33%–64% at 24 months (Rousselot et al. 2014; Lee et al. 2016; Etienne et al. 2017); in dasatinib, discontinuation studies were 48% at 12 months and 46% at 24 months (Shah et al. 2020), and in nilotinib, discontinuation studies were 51.6–58% at 12 months and 49–53% at 24 months (Hochhaus et al. 2017a, b; Mahon et al. 2018; Ross et al. 2018). Therefore, *TKIs combined with RIF may be an efficacy treatment strategy for patients with CP-CML who would be willing to attempt discontinuation.*

*PFS and OS rates remain high and comparable between the trial groups (more than 90%).* These results are consistent with the long-term outcomes in patients with newly diagnosed CP-CML who received the second-generation TKIs nilotinib, dasatinib, and bosutinib (Cortes et al. 2016; Hochhaus et al. 2016a, b; Brümmendorf et al. 2022). Patients with CML treated with TKI can expect a near-normal life expectancy (Cortes et al. 2021).

*Safety data were consistent with the known AE profiles of RIF in newly diagnosed patients with APL and imatinib in newly diagnosed patients with CP-CML* (Preudhomme et al. 2010; Zhu et al. 2018, Chen, Zhu et al. 2021). Most AEs occurred primarily during the first six months of treatment and were generally controllable, which is consistent with other trials (Zhu et al. 2018, Chen et al. 2021). No severe adverse effects were observed at a cumulative dose of 105,840 mg realgar in this study. The predominant AEs of the RIF plus imatinib regimen were diarrhea, vomiting, nausea, edema, upper respiratory infection, cytopenia, and liver function abnormalities; most participants tolerated the regimen well and did not need to adjust the drug dose in our trial. The incidence and duration of hematological AEs in the two groups were similar, implying that RIF did not significantly increase hematological toxicity. The incidence of hepatotoxicity in the combination group was similar to that reported in other studies involving arsenic agents (Zhu et al. 2018, Chen et al. 2021), though all being grade 1 or grade 2 in our trial. It has been reported that a small prolongation of the QTc interval occurred in patients who received arsenic trioxide, however, to a lesser extent in patients who received RIF, which is consistent with previous reports (Lo-Coco et al. 2013; Zhu et al. 2018).

The combination therapy was more toxic than imatinib alone regarding gastrointestinal side effects, such as diarrhea (*p* = 0.036), vomiting (*p* = 0.004), nausea (*p* = 0.006), and liver function abnormalities, including ALT (*p* = 0.006) and AST (*p* < 0.001), which led to temporary interruptions and/or reductions in the combination group (most cases stopped both RIF and imatinib) (Table S6). *The rates of MMR and MR*^*4*^* in the combination group were lower than the imatinib group in the early stages of our trial; however, a reversal emerged in response between the trial groups at 9 months when AEs decreased. Furthermore, the difference between combination therapy and imatinib increased subsequently over time, and at 12 months, the MR*^*4.5*^* rate showed a significant increase in the combination arm (p* = *0.043). In addition, the reasons for the more frequent occurrence of muscle cramps (p* = *0.003), rash (p* = *0.086), pruritus, muscle ache, and bleeding with imatinib alone might be due to dose interruptions and/or reductions and gastrointestinal symptoms affecting drug absorption at the early stage in the combination group.* This also supports our speculation that the time of occurrence of hematological AEs in the combination group lagged behind that in the imatinib group. Therefore, an early molecular response might not reflect the efficacy of the two arms well.

## Limitations

This study had several limitations. First, the significant limitation of our study was low enrollment due to recruitment termination during the COVID-19 pandemic in early 2020, which underpowered the result. Second, our trial found a higher rate of achieving MR^4.5^ in participants who received RIF plus imatinib than those who received imatinib monotherapy. Thus, in future, we could plan to design a trial to further evaluate RIF plus TKI for this index. Third, the follow-up time is a little short for TFR, which is another limitation of our trial, and we will continue to follow these patients for a long time.

In summary, there was no statistically significant difference in MMR rates between combination and imatinib arms at 6 months. However, we found that the combination regimen may increase the rates of **MR**^**4.5**^ which are a prerequisite for TFR, than imatinib alone in participants with de novo CP-CML. In addition, the safety profile of combination treatment was similar to that of imatinib. As enrollment in this trial was terminated early, the efficacy of RIF with imatinib in this setting remains to be established.

## Supplementary Information

Below is the link to the electronic supplementary material.

Supplementary file1 (PDF 245 KB)

Supplementary file2 (PDF 95 KB)

## Data Availability

The datasets generated during and/or analyzed during the current study are available from the corresponding author on reasonable request.
