# Supplementary material for: Oral arsenic plus imatinib versus imatinib solely for newly diagnosed chronic myeloid leukemia: a randomized phase 3 trial with 5-year outcomes
Source: J Cancer Res Clin Oncol. 2024 Apr 11;150(4):189. doi: 10.1007/s00432-024-05700-x (PMC11009770; doi:10.1007/s00432-024-05700-x)
Supplement: Supplementary file 1 — Supplementary file1 (PDF 245 KB) [file 432_2024_5700_MOESM1_ESM.pdf]

Supplementary Material

Table S1. Study Inclusion and Exclusion Criteria

Inclusion criteria	● Age≥18 years and ≤75years<br>● Cytogenetic diagnosis of Ph-positive CP CML within 12 months, satisfied all of the following:<br>• Bone marrow cytogenetics Ph chromosome t (9;22) positive (check at least 20 mitotic figures) and/or molecular biology test *BCR::ABL* fusion gene positive<br>• Blasts<15%, basophils<20%, and blasts promyelocytes<30% in peripheral blood and bone marrow<br>• Platelets > 100×10^{9}/L, or ≤100×10^{9}/L related to prior medical treatement<br>• No extramedullary disease (except liver or spleen)<br>● ECOG performance status of 0 or 2<br>● Adequate hepatic function (ALT/AST≤2.5×ULN, total bilirubin≤1.5×ULN)<br>● Adequate renal function (creatinine≤1.5×ULN)<br>● Serum amylase and lipase≤1.5×ULN<br>● Signed and dated informed consent form prior to protocol-specific screening procedures
Exclusion criteria	● Prior or undergoing antileukemia treatment, included any one of the following:<br>• ≤2 days of hydroxyurea or ≤7 days of anagrelide<br>• ≤14 days of Homoharringtonine<br>• ≤28 days of Cytarabine, interferon-alpha, anthracyclines, mitoxantrone, or etoposide<br>• prior any form of tyrosine kinase inhibitor or arsenic treatment<br>• prior stem-cell transplantation<br>● Ph-negative CML or history of AP or BP CML<br>● CML with chronic myelofibrosis<br>● Major surgery within 4 weeks of random assignment<br>● CNS leukemia<br>● Impaired cardiac function including any one of the following:<br>• Echocardiogram with left ventricular ejection fraction (LVEF) <50%<br>• Screening ECG with a QTc >450 msec in men or QTc >470 msec in women<br>• Patients with congenital long QT syndrome<br>• clinically significant ventricular arrhythmia<br>• Clinically significant bradycardia (heart rate< 50 beats/min)<br>• Congestive heart failure (NY Heart Association class III or IV)<br>• Complete left bundle branch block<br>• History of myocardial infarction or unstable angina pectoris within 12 months<br>• unexplained syncope<br>● Unstable or severe uncontrolled medical condition, evidence of serious active infection, uncontrolled hypertension, significant psychiatric illness, or any important medical illness or abnormal laboratory finding that would, in the investigator's judgment, increase the risk associated with the patient's participation in the study<br>● History of acute pancreatitis within 1 year or history of chronic pancreatitis

	● Recent or ongoing clinically significant GI disorder<br>● Pregnant or breastfeeding<br>● Participated in other new drug clinical trials as subjects within 4 weeks<br>● Allergic to any test drugs and its excipients<br>● Patients with any significant history of non-compliance to medical regimens or with inability to grant a reliable informed consent

Abbreviations: AP, accelerated phase; BP, blast phase; CML, chronic myeloid leukemia; CP, chronic phase; ECOG, Eastern Cooperative Oncology Group; Ph, Philadelphia chromosome; QTc, corrected QT interval; ULN, upper limit of normal.

Procedures

Treatment, dose modification and measure

Following randomization, patients assigned to combination group received RIF (target dose, 65 mg per square meter of body-surface area per day in three oral divided doses), and patients in imatinib group received placebo (RIF simulant) for 14 consecutive days every month. All patients also received 400 mg imatinib orally daily. Gradually escalating doses of RIF or placebo were given in the first month of treatment, until up to the target dose as long as grade 3 or 4 toxicity did not occur. From the second course on, RIF or placebo was administered on the target dose directly. Total course of treatment is 12 months (a month defined as 28 days). After 12 months, all patients only received imatinib daily.

Therapy continued until treatment failure, unacceptable toxicity, or other criteria for withdrawal were met. Doses could be reduced to manage drug-related adverse events (AE) and re-escalated once events resolved. Dose reductions to less than 2250 mg for RIF or placebo, or less than 300 mg for imatinib were not permitted. An escalation of imatinib dose was not permitted.

Molecular response was assessed for *BCR::ABL1* performed by quantitative real-time polymerase-chain-reaction (qRT-PCR) from peripheral blood, using *ABL1* as control gene, expressed on the international scale (IS) (Müller, Cross et al. 2009) with conversion factor of 0.66, assayed at a centralized laboratory (Guangzhou KingMed Diagnostics Group Co.) at baseline, every 3 months for the first year, and every 3 or 6 months thereafter. Bone marrow cytogenetic analyses were conducted at screening and at months 3, 6 and 12. Blood and urinary arsenic concentration were measured at screening and at months 6 and 12, then every year up to 3 years from randomization. Other laboratory assessments (including complete blood counts, serum chemistry, blood sugar, serum potassium, calcium, and magnesium) were measured at screening, baseline, at weeks 2 for the first 3 months and the last day of every month thereafter.

Table S2. Patient Status at 5 Years.

Status	RIF/IM(n=96)	Placebo/IM(n=95)
Remained on study	80(83.3)	79(83.2)
Remained on core treatment	45(46.9)	44(46.3)
Exchange to other anti-CML drugs*	35(36.5)	35(36.8)
Suboptimal response/treatment failure	27 (28.1)	25(26.3)
Disease progression to AP/BP*	6(6.2)	4(4.2)
Adverse event	7 (7.3)	9(9.5)
Other	1(1.0)	1(1.1)
Death	7(7.3)	5(5.3)
CML related	4(4.2)	2(2.1)
Car accident	1(1.0)	0
Other tumor	0	1(1.1)
Cerebral hemorrhage	0	1(1.1)
Unknown	2(2.1)	1(1.1)
Lost to follow-up	6(6.3)	9(9.5)
Other	3(3.1)	1(1.1)

NOTE. Data are No.(%). * Other anti-CML drugs were the second-generation TKIs in this study. Abbreviations: RIF,realgar-Indigo naturalis formula; IM,Imatinib; CML, chronic myeloid leukemia; AP, accelerated phase; BP, blast phase.

Table S3. Five-year outcomes according to Sokal risk score.

	RIF/IM	Placebo/IM
Low Sokal risk,n	45	44
$MR^{4.5}$ by 5 years,n(%)	32(71.1)	28(63.6)
$MR^{4.5}$ by 2 years,n(%)	23(51.1)	16(36.4)
Estimated 5-year PFS on study,%[a]	100	97.7
Estimated 5-year OS on study,%[a]	100	97.7
Intermediate Sokal risk,n	38	38
$MR^{4.5}$ by 5 years,n(%)	23(60.5)	21(55.3)
$MR^{4.5}$ by 2 years,n(%)	11(28.9)	13(34.2)
Estimated 5-year PFS on study,%[a]	86.8	94.7
Estimated 5-year OS on study,%[a]	86.5	92.1
High Sokal risk,n	13	13
$MR^{4.5}$ by 5 years,n(%)	4(30.8)	7(53.8)
$MR^{4.5}$ by 2 years,n(%)	3(23.1)	5(38.5)
Estimated 5-year PFS on study,%[a]	92.3	92.3
Estimated 5-year OS on study,%[a]	81.8	90.9

Abbreviations: AP/BC, accelerated phase/blast crisis; $MR^{4.5}$, molecular response 4.5 (*BCR::ABL*≤0.0032% on the International Scale); OS,overall survival; PFS,progression-free survival.a,Estimated by Kaplan–Meier analysis.

Table S4. Safety findings

Event	RIF/IM(n=96)		Placebo/IM(n=95)	
	Any grade(n,%)	Grade 3/4(n,%)	Any grade(n,%)	Grade 3/4(n,%)
All events	93(96.9)	34(35.4)	87(91.6)	38(40.0)
Nonhematologic adverse event	88(91.7)	6(6.3)	83(87.4)	6(6.3)
Rash	6(6.3)	0(0.0)	13(13.7)	2(2.1)
Pruritus	9(9.4)	0(0.0)	12(12.6)	0(0.0)
Constipation	2(2.1)	0(0.0)	1(1.1)	0(0.0)
Diarrhea	26(27.1)	0(0.0)	14(14.7)	0(0.0)
Vomiting	25(26.0)	1(1.0)	17(17.9)	0(0.0)
Nausea	33(34.4)	1(1.0)	17(17.9)	1(1.1)
Muscle ache	25(26.0)	0(0.0)	32(33.7)	0(0.0)
Muscle cramps	2(2.1)	0(0.0)	12(12.6)	0(0.0)
Abdominal pain	19(19.8)	0(0.0)	12(12.6)	0(0.0)
Abdominal distension	14(14.6)	0(0.0)	9(9.5)	0(0.0)
Fatigue	14(14.6)	0(0.0)	10(10.5)	0(0.0)
Bone pain	5(5.2)	0(0.0)	4(4.2)	0(0.0)
Joint pain	9(9.4)	0(0.0)	8(8.4)	0(0.0)
Headache	9(9.4)	0(0.0)	7(7.4)	0(0.0)
Edema*	65(67.7)	2(2.1)	57(60.0)	0(0.0)
Facial edema	58(60.4)	2(2.1)	50(52.6)	0(0.0)
Pyrexia	12(12.5)	0(0.0)	13(13.7)	1(1.1)
Pulmonary infection	1(1.0)	0(0.0)	1(1.1)	0(0.0)
Upper respiratory infection	38(39.6)	1(1.0)	28(29.5)	0(0.0)
Urinary infection	6(6.3)	1(1.0)	8(8.4)	0(0.0)
Bleeding	5(5.2)	0(0.0)	11(11.6)	1(1.1)
Cytopenia	71(74.0)	30(31.3)	62(65.3)	26(27.4)
Anemia	43(44.8)	5(5.2)	36(37.9)	7(7.4)
Thrombocytopenia	34(35.4)	15(15.6)	38(40.0)	13(13.7)
Leucopenia	64(66.7)	23(24.0)	52(54.7)	17(17.9)
Laboratory abnormality	59(61.5)	4(4.2)	52(54.7)	5(5.3)
Elevated ALT	32(33.3)	0(0.0)	12(12.6)	1(1.1)
Elevated AST	30(31.3)	0(0.0)	10(10.5)	1(1.1)
Elevated alkaline phosphatase	15(15.6)	0(0.0)	16(16.8)	0(0.0)
Elevated GGT	8(8.3)	0(0.0)	10(10.5)	1(1.1)
Elevated bilirubin	1(1.0)	0(0.0)	1(1.1)	1(1.1)
Hypofibrinogenaemia	6(6.3)	1(1.0)	5(5.3)	0(0.0)

Elevated amylase	8(8.3)	0(0.0)	8(8.4)	0(0.0)
Elevated lipase	2(2.1)	0(0.0)	0(0.0)	0(0.0)
Hyperlipemia	5(5.2)	0(0.0)	5(5.3)	0(0.0)
Hyperglycemia	7(7.3)	0(0.0)	13(13.7)	0(0.0)
Hyperuricemia	6(6.3)	0(0.0)	1(1.1)	0(0.0)
Elevated serum creatinine	3(3.1)	0(0.0)	1(1.1)	0(0.0)
Elevated LDH	7(7.3)	0(0.0)	11(11.6)	0(0.0)
Elevated creatine kinase	2(2.1)	0(0.0)	3(3.2)	0(0.0)
Hypocalcemia	6(6.3)	0(0.0)	5(5.3)	0(0.0)
Hypokalemia	3(3.1)	0(0.0)	9(9.5)	1(1.1)
Hypophosphatemia	25(26.0)	3(3.1)	19(20.0)	2(2.1)
Hypomagnesemia	2(2.1)	0(0.0)	1(1.1)	0(0.0)

Data are number of patients(%). Adverse events were graded according to the Common Toxicity Criteria of the National Cancer Institute.* Edema includes edema, eye edema, orbital and periorbital edema, face edema, localized edema, edema peripheral, allergic edema, weight increased, pharyngeal edema, and pulmonary edema. Abbreviations: RIF,realgar-Indigo naturalis formula; IM,Imatinib; ALT,alanine aminotransferase; AST,aspartate aminotransferase; ALP,alkaline phosphatase; GGT,γ-glutamyl transpeptidase.

Table S5. Drug-related cardiac adverse events.

Event	RIF/IM(n=96)		Placebo/IM(n=95)	
	Any grade(n,%)	Grade 3/4(n,%)	Any grade(n,%)	Grade 3/4(n,%)
All events	12(12.5)	0(0.0)	10(10.5)	1(1.1)
Atrioventricular block	1(1.0)	0(0.0)	1(1.1)	0(0.0)
Prolonged QT interval*	0(0.0)	0(0.0)	4(4.2)	1(1.1)
Shortened QT interval	0(0.0)	0(0.0)	1(1.1)	0(0.0)
T-wave changes	2(2.1)	0(0.0)	3(3.2)	0(0.0)
Sinus bradycardia	4(4.2)	0(0.0)	0(0.0)	0(0.0)
Sinus tachycardia	2(2.1)	0(0.0)	2(2.1)	0(0.0)
Sinus arrhythmia	1(1.0)	0(0.0)	1(1.1)	0(0.0)
Auricular premature beat	3(3.1)	0(0.0)	2(2.1)	0(0.0)
Ventricular premature beat	1(1.0)	0(0.0)	0(0.0)	0(0.0)

Data are number of patients(%). Adverse events were graded according to the Common Toxicity Criteria of the National Cancer Institute. * The results of prolonged QTc interval corrected for heart rate were 461,469,508 and 510msec.

Table S6. Dose interruptions and reductions for adverse events.

Event(n,%)	RIF/IM(n=96)	Placebo/IM(n=95)
All events	45(46.9)	34(35.8)
Gastrointestinal abnormality	8(8.3)	2(2.1)
Hepatic toxic effects	10(10.4)	3(3.2)
Hematologic abnormality	23(24.0)	21(22.1)
Cardiac abnormality	1(1.0)	4(4.2)
Rash	4(4.2)	5(5.3)
Infection	4(4.2)	3(3.2)
Others	13(13.5)	8(8.4)

Data are number of patients(%). Adverse events were graded according to the Common Toxicity Criteria of the National Cancer Institute.Abbreviations: RIF,realgar-Indigo naturalis formula; IM,Imatinib.

Reference

Müller, M. C., et al. (2009). "Harmonization of molecular monitoring of CML therapy in Europe." Leukemia **23**(11): 1957-1963.
