# Supplementary material for: Oral arsenic plus imatinib versus imatinib solely for newly diagnosed chronic myeloid leukemia: a randomized phase 3 trial with 5-year outcomes
Source: J Cancer Res Clin Oncol. 2024 Apr 11;150(4):189. doi: 10.1007/s00432-024-05700-x (PMC11009770; doi:10.1007/s00432-024-05700-x)
Supplement: Supplementary file 2 — Supplementary file2 (PDF 95 KB) [file 432_2024_5700_MOESM2_ESM.pdf]

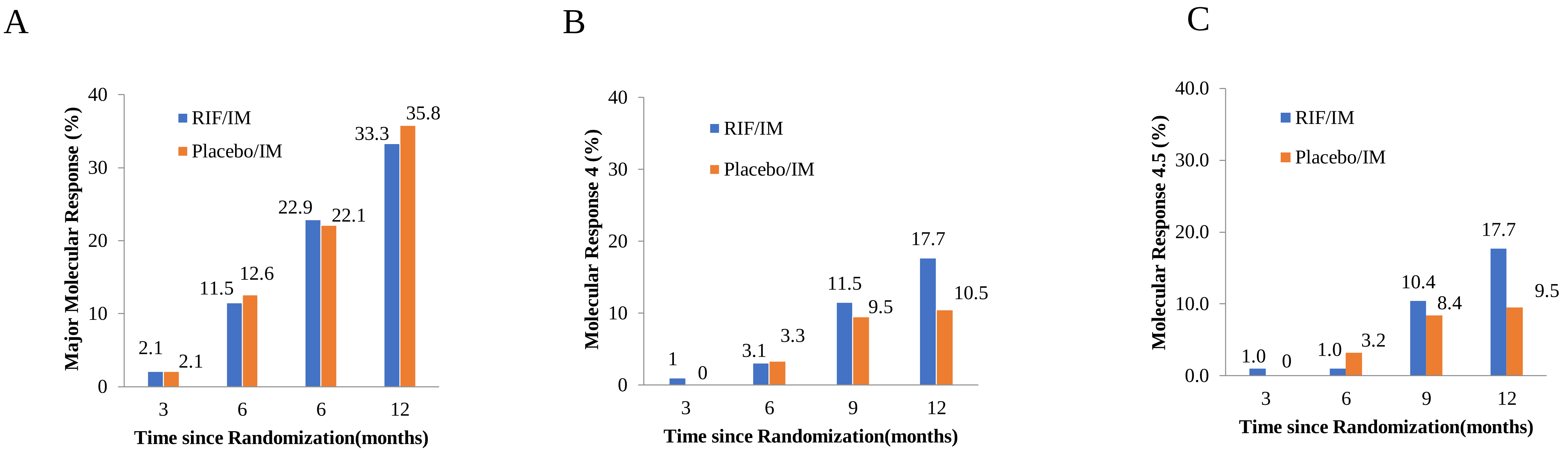


Fig S1. Rates of Molecular Response at 3, 6, 9, and 12 Months. The results in the intention-to-treat population were calculated by means of the Cochran–Mantel–Haenszel test, stratified according to the Sokal risk group, after the last patient had completed 12 cycles of therapy (with a 28 day duration for each cycle). The rates of (A) major molecular response (MMR; *BCR::ABL*$^{IS}$≤0.1%), (B) molecular response 4 ($MR^4$; *BCR::ABL*$^{IS}$≤ 0.01%) and (C) molecular response 4.5 ($MR^{4.5}$; *BCR::ABL*$^{IS}$≤0.0032%) at 3, 6, 9 and 12 months are shown. IS, International Scale.

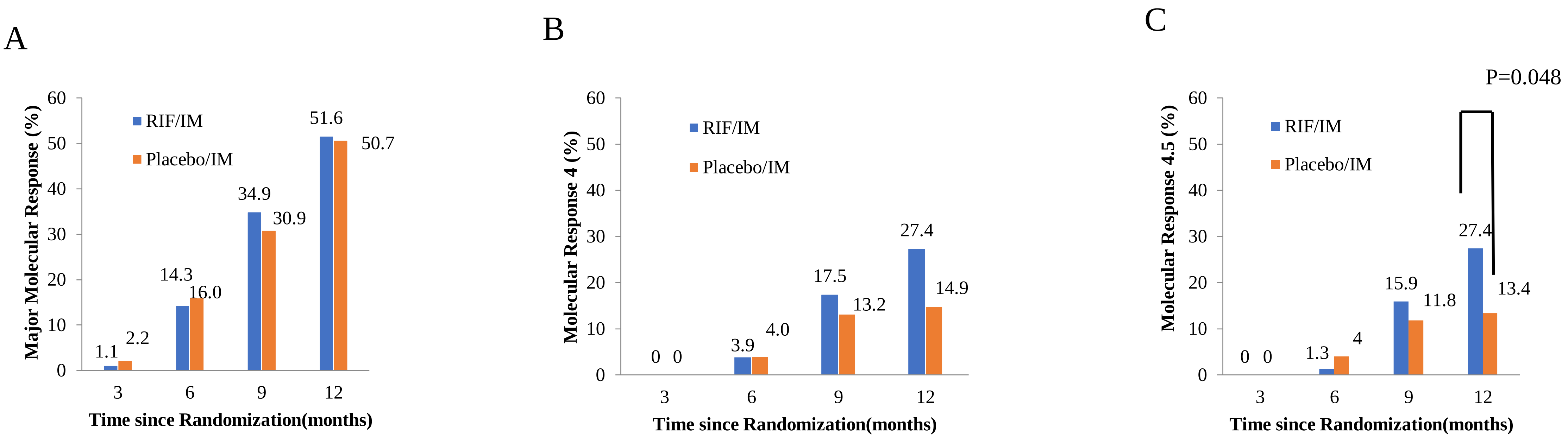


Fig S2. Rates of Molecular Response at 3, 6, 9, and 12 Months. The results in the per protocol population were calculated by means of the Cochran–Mantel–Haenszel test, after the last patient had completed 12 cycles of therapy. The rates of (A) major molecular response (MMR; $BCR::ABL^{IS} \leq 0.1\%$), (B) molecular response 4 ($MR^4$; $BCR::ABL^{IS} \leq 0.01\%$) and (C) molecular response 4.5 ($MR^{4.5}$; $BCR::ABL^{IS} \leq 0.0032\%$) at 3, 6, 9 and 12 months are shown. IS, International Scale.

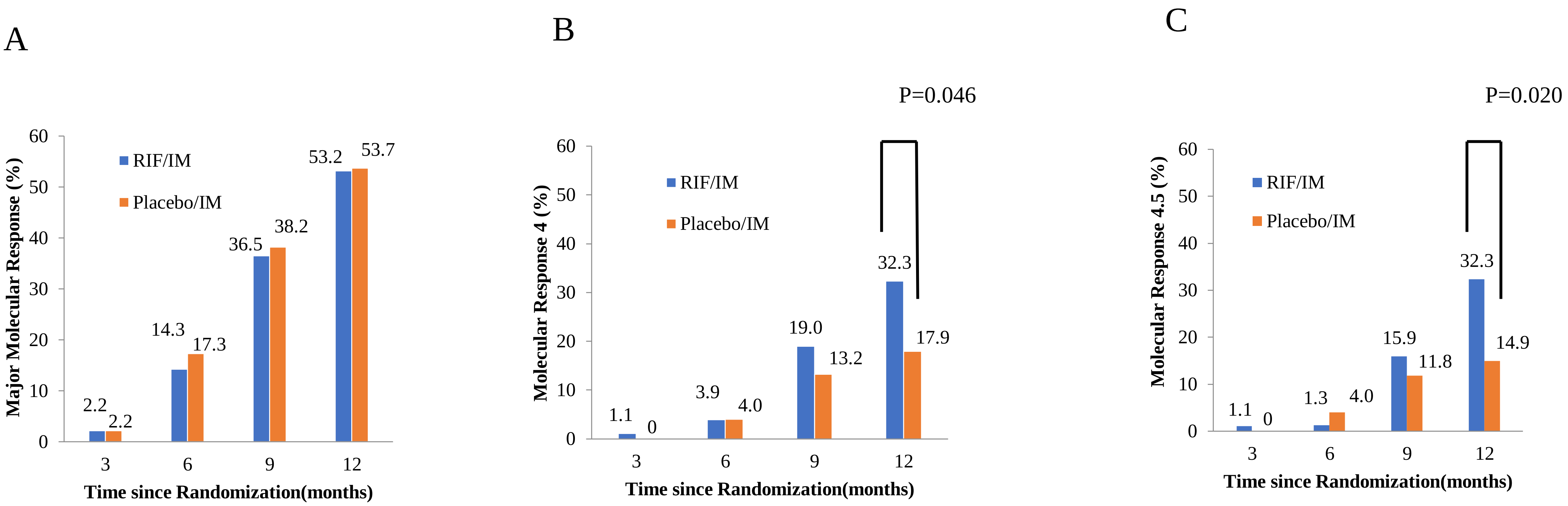


Fig S3. Cumulative molecular response rates. The results in the per protocol population were calculated by means of the Cochran–Mantel–Haenszel test. Cumulative proportion of patients with (A) major molecular response (MMR; *BCR::ABL*$^{IS}$≤0.1%), (B) molecular response 4 (MR$^{4}$; *BCR::ABL*$^{IS}$≤ 0.01%) and (C) molecular response 4.5 (MR$^{4.5}$; *BCR::ABL*$^{IS}$≤0.0032%) by 3, 6, 9 and 12 months are shown. IS, International Scale.

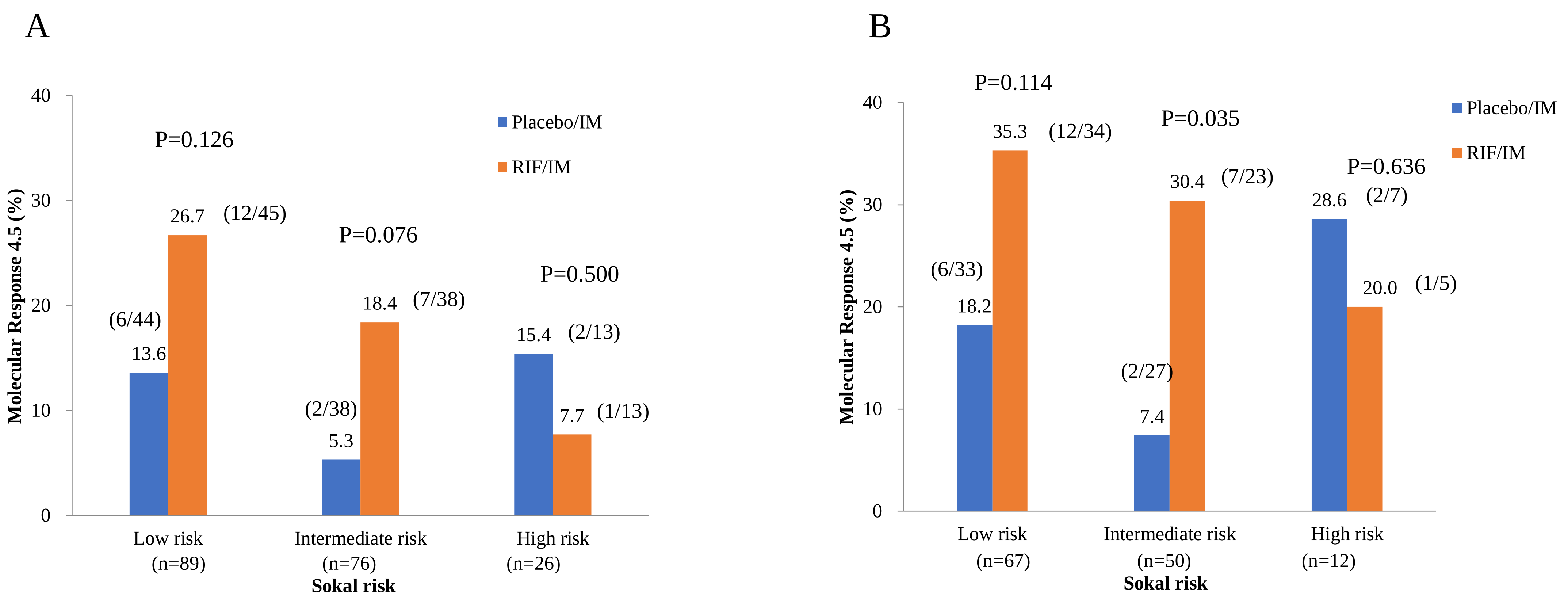


Fig S4. (A) and (B) molecular response 4.5 (MR$^{4.5}$; *BCR::ABL1*$^{IS}$ ≤0.0032%) by 12 months by Sokal risk score in the intention-to-treat population and per protocol population,  respetively. IS, International Scale.
